# Surgical treatment of benign splenic lesions in pediatric patients: a case series of 30 cases from a single center

**DOI:** 10.1186/s12893-022-01745-2

**Published:** 2022-07-29

**Authors:** Zengmeng Wang, Chunhui Peng, Dongyang Wu, Kai Wang, Jiatong Xu, Jihang Sun, Wenbo Pang, Cailin Ding, Yajun Chen

**Affiliations:** 1 General Surgery Department, Beijing Children’s Hospital, Capital Medical University, National Center for Children’s Health, 56# Nanlishi Road, Beijing, 100045 China; 2 Pathology Department, Beijing Children’s Hospital, Capital Medical University, National Center for Children’s Health, Beijing, China; 3 Radiology Department, Beijing Children’s Hospital, Capital Medical University, National Center for Children’s Health, Beijing, China

**Keywords:** Spleen benign lesion, Splenectomy, Pathology, Imaging, Pediatric, Prognosis

## Abstract

**Background:**

Benign splenic lesions are rarely encountered. This study aimed to review the clinical characteristics and surgical outcomes in a case series of 30 pediatric patients.

**Methods:**

From January 1st, 2001 to December 31st, 2021, 30 pediatric patients from a single center were consecutively included. Electronic medical records were reviewed and patients were followed up. Clinical presentations, imaging features, surgical procedures, pathological diagnoses, and prognoses were summarized. The lesion locations and 7-day postoperative platelet levels were compared between total and partial splenectomy patients.

**Results:**

Eighteen males and twelve females were included, with mean age at surgery 116.4 ± 43.6 months. The clinical presentations included abdominal pain (16/30), splenomegaly (6/30), skin petechia (2/30), hemolytic jaundice (1/30), and no symptoms (5/30). Pathological diagnoses included congenital epithelial cyst (CEC, 17/30), vascular malformation (8/30), sclerosing angiomatoid nodular transformation (SANT, 3/30), hamartoma (1/30), and leiomyoma (1/30). Patients undergone total splenectomy were more likely to have a lesion involving the hilum than those undergone partial splenectomy (68.4% vs 31.6%, *P* = 0.021). The 7-day postoperative platelet level was higher in total splenectomy patients than partial splenectomy patients (adjusted means 694.4 × 10^9^/L vs 402.4 × 10^9^/L, *P* = 0.002).

**Conclusions:**

Various clinical characteristics of pediatric benign splenic lesions are summarized. The most common pathological diagnoses are congenital epithelial cyst and vascular malformation. Partial and total splenectomy result in good prognosis with a low recurrence rate, and the former is preferred to preserve splenic function if possible.

## Background

Benign splenic lesions are rare, especially in children [1, 2]. Both cystic and solid lesions of a wide histological spectrum can be encountered [3]. Different treatment strategies are reported, including spleen preserving or non-preserving methods [2, 4]. For diagnostic and treatment purposes surgical interventions are usually needed via open or laparoscopic/robotic approaches [5, 6]. Currently published articles are generally adult cases with limited patient numbers, and reports of pediatric cases are even less. In this research we present our clinical experience in this rare entity, based on a case series of 30 patients over 21 years from a single tertiary pediatric center. This retrospective study mainly focuses on clinical features and surgical outcomes, to add pediatric clinical evidence to this rare entity.

## Methods

### Study design

This is a retrospective case series of 30 consecutive patients from a single tertiary pediatric center. From January 1st, 2001 to December 31st, 2021, all patients diagnosed as benign splenic lesion and surgically treated were included. Diagnoses were established on imaging study demonstrating a splenic lesion, including computed tomography, magnetic resonance imaging, and ultrasonography. Patients with pathological evidence of malignancy (original, metastatic, or infiltrated) were excluded. Follow-ups were performed by outpatient visits.

### Data collection and grouping

The following data were collected by reviewing inpatient and outpatient electronic medical records: clinical history, laboratory results, imaging results, surgical procedures, pathological diagnoses, and prognoses. Imaging results were reviewed to determine the location of the lesion inside the spleen. The spleen was anatomically divided into upper pole, hilum, and lower pole, and the lesion could involve one or more parts. According to different surgical procedures, all patients were divided into total splenectomy group and partial splenectomy group. Postoperative complications such as overwhelming post-splenectomy infection, arterial and venous thrombotic events, and pulmonary hypertension, were checked during follow-ups.

### Statistical analyses

Continuous variables were described with means with standard deviations or medians with quartiles according to data distribution, and categorical variables were described with percentages. The rates of lesion involving the splenic hilum were compared between total and partial splenectomy groups using Fisher’s exact test. The pre- and postoperative platelet levels were compared with paired sample t test. The postoperative platelet levels were compared between total and partial splenectomy groups with analysis of covariance using preoperative platelet level as a covariate. A *P* value < 0.05 was considered statistically significant. Data was analyzed with IBM SPSS Statistics 26.0.

## Results

### General information and follow-up results

**Fig. 1:**
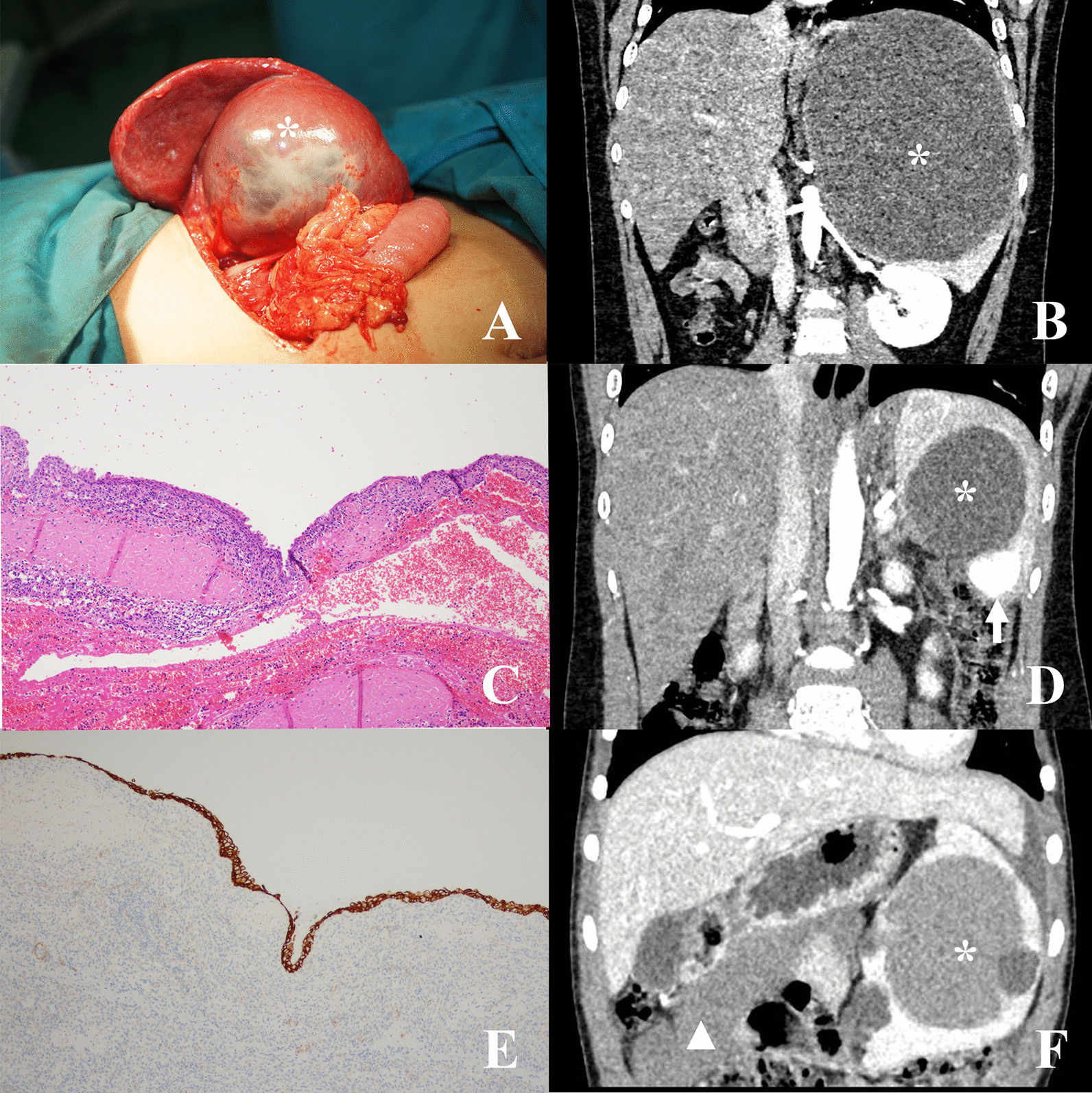
Splenic CECs (“*” marks the cysts). **A** A recurrent cyst after unroofing (Case 21). **B** The cyst compressing left renal and renal artery (Case 26). **C** Stratified squamous epithelium (HE staining). **D** Simultaneous separate CEC and hemagioma (white arrow shows the enhanced hemangioma, Case 19). **E** Cytokeratin immunohistochemical staining labels the epithelium. **F** Simultaneous separate CEC and omentum mesothelial cyst (white triangle shows the omentum mesothelial cyst, Case 28)

This case series consisted of 30 patients diagnosed as benign splenic lesions, including 18 males and 12 females. The detailed clinical information of each case is demonstrated in Table 1. The mean age at surgery was 116.4 ± 43.6 months. Three patients (case 11, 12, 21) were diagnosed as recurrent splenic cyst after previous surgical unroofing at other centers (Fig. 1A), and the previous procedures were not analyzed in this study. The median length of follow-up was 45.4 (18.7–85.7) months. No recurrence, overwhelming post splenectomy infection, arterial or venous thrombotic events, or pulmonary hypertension occurred in all patients during follow-ups. One patient (Case 26) experienced focal splenic infarction shortly after partial splenectomy, and recovered without any intervention in 6 months, maintaining a functional residual spleen with adequate blood supply.

**Table 1:** Detailed clinical information of 30 cases

No.	Sex	Age (Mon)	Clinical presentation	Lesion size (cm)	Lesion feature	Pathology	Surgery	Addition
1	M	72.3	Abdominal pain	7.0 × 6.5 × 5.5	Focal Cystic H	CEC	O + T	
2	M	147.5	Hemolytic anemia with jaundice	7.5 × 6.0 × 5.0	Focal Solid Lo	hamartoma	O + T	
3	F	122.1	Abdominal pain	5.5 × 5.0 × 5.0	Focal Solid U + H	SANT	O + P	
4	M	66.8	Abdominal pain	3.9 × 3.8 × 3.1	Focal Cystic U + H	CEC	O + P	
5	M	196.3	Splenomegaly	13.0 × 11.0 × 8.0	Focal Cystic Lo	CEC	O + P	
6	F	69.5	Abdominal pain	4.5 × 3.5 × 3.2	Focal Solid H	SANT	O + T	
7	F	85.2	Abdominal pain	4.5 × 4.5 × 4.3	Focal Solid H	Leiomyoma	O + T	
8	M	109.1	Abdominal pain	4.5 × 4.0 × 3.2	Focal Solid Lo	SANT	O + T	
9	M	114.3	Abdominal pain	7.5 × 7.5 × 7.5	Focal Cystic H	CEC	O + T	
10	M	93.8	Skin petechia	10.0 × 6.0 × 2.5	Diffused Cystic + Solid U + H + Lo	Lymphangio-hemangiomatosis	O + T	Consumptive coagulopathy
11	M	176.7	No symptom	9.2 × 8.8 × 7.7	Focal Cystic U	CEC	O + P	Recurrent case after unroofing
12	M	176.1	No symptom	17.0 × 16.0 × 13.0	Focal Cystic U	CEC	O + P	Recurrent case after unroofing
13	M	102.6	No symptom	5.1 × 4.0 × 3.4	Focal Solid Lo	Lymphangioma	O + P	
14	F	169.4	Abdominal pain	8.9 × 7.7 × 7.1	Focal Cystic U + H	CEC	L + T	
15	M	48.1	Abdominal pain	7.3 × 6.6 × 6.6	Focal Cystic U	CEC	O + P	
16	M	144.3	Abdominal pain	15.1 × 15.1 × 12.3	Focal Cystic U + H	CEC	O + P	
17	F	103.3	No symptom	7.5 × 6.8 × 5.9	Focal Solid H	Lymphangioma	L + T	
18	F	151.1	Splenomegaly	5.3 × 5.0 × 4.9	Focal Solid H + Lo	Lymphangioma	O + P	
19	M	144.7	Abdominal pain	6.6 × 6.5 × 5.4	Multifocal Cystic + Solid U	CEC + hemangioma	O + P	Simultaneous separate CEC and hemangioma
20	F	28.6	Splenomegaly	16.0 × 10.0 × 5.0	Diffused Solid U + H + Lo	Lymphangiomatosis	O + T	
21	F	149.4	Abdominal pain	8.0 × 7.6 × 6.4	Multifocal Cystic U + H	CEC	O + T	Recurrent case after unroofing
22	M	79.3	No symptom	8.1 × 7.7 × 7.3	Focal Cystic U	CEC	O + T	
23	F	97.8	Splenomegaly	22.4 × 13.6 × 6.9	Focal Cystic H	Lymphangioma	O + T	Involving omentum at splenic hilum
24	M	130.4	Abdominal pain	10.7 × 10.4 × 5.7	Focal Cystic U + H	CEC	O + T	
25	F	56.7	Skin petechia	20.0 × 15.0 × 8.0	Diffused Solid U + H + Lo	Lymphangio-hemangiomatosis	O + T	Consumptive coagulopathy
26	M	144.7	Splenomegaly	19.9 × 17.4 × 15.1	Focal Cystic U	CEC	L + P	Postoperative focal spleen infarction
27	M	151.4	Splenomegaly	15.1 × 13.9 × 8.0	Multifocal Cystic U + H + Lo	CEC	L + T	
28	M	96.1	Abdominal pain	12.9 × 10.4 × 9.5	Multifocal Cystic U + H + Lo	CEC	O + T	simultaneous omental mesothelial cyst
29	F	179.5	Abdominal pain	18.5 × 15.3 × 13.4	Focal Cystic U + H	CEC	L + P	
30	F	83.7	Abdominal pain	6.2 × 4.9 × 4.4	Focal Solid U	Lymphangioma	L + P	focal endothelial papillary projections

*M* male, *F* female, *U* upper pole, *H* hilum, *Lo* lower pole, *CEC* congenital epithelial cyst, *SANT* sclerosing angiomatoid nodular transformation, *O* open, *L* laparoscopic, *T* total splenectomy, *P* partial splenectomy

### Clinical presentations

Sixteen (51.6%) patients manifested as abdominal pain in the left hypochondrium area, 6 (20.0%) patients manifested as abdominal mass (splenomegaly), 2 (6.7%) patients manifested as skin petechia, 1 (3.3%) patient manifested as hemolytic anemia with jaundice, and 5 (16.7%) patients were asymptomatic.

The skin petechia was caused by consumptive coagulopathy in two patients (Case 10 and 25), who were both finally diagnosed as splenic diffused lymphangiohemagiomatosis. In both patients the consumptive coagulopathy could intermittently improve spontaneously. One patient (Case 2) manifested as hemolytic anemia with jaundice and was finally diagnosed as splenic hamartoma.

## Pathological diagnoses and imaging results

Final postoperative pathological diagnoses included 17 CECs (56.7%), 8 vascular malformations (26.7%), 3 SANTs (10%), 1 hamartoma (3.3%), and 1 leiomyoma (3.3%).

### CEC

Seventeen patients fell into this category. Some large cysts could compress the kidney and its artery (Fig. 1B). One patient (Case 19) had a simultaneous separate hemangioma (Fig. 1D), and another one (Case 28) had a simultaneous omental mesothelial cyst (Fig. 1F). The CECs had different types of epithelial lining based on the combination of three basic epithelial types: unilayered cuboidal/low columnar mesothelial epithelium, stratified cuboidal transitional epithelium, and nonkeratinizing stratified squamous epithelium (Fig. 1C). Different epithelium types of the 17 CEC cases included 8 squamous, 5 mesothelial, 2 mesothelial + squamous, and 2 transitional + squamous epithelium. There were 12 cases contained squamous epithelium, and 4 of them had focal loss of epithelial lining. Cytokeratin immunohistochemical staining results were positive in CECs (Fig. 1E).

### Vascular malformation

**Fig. 2:**
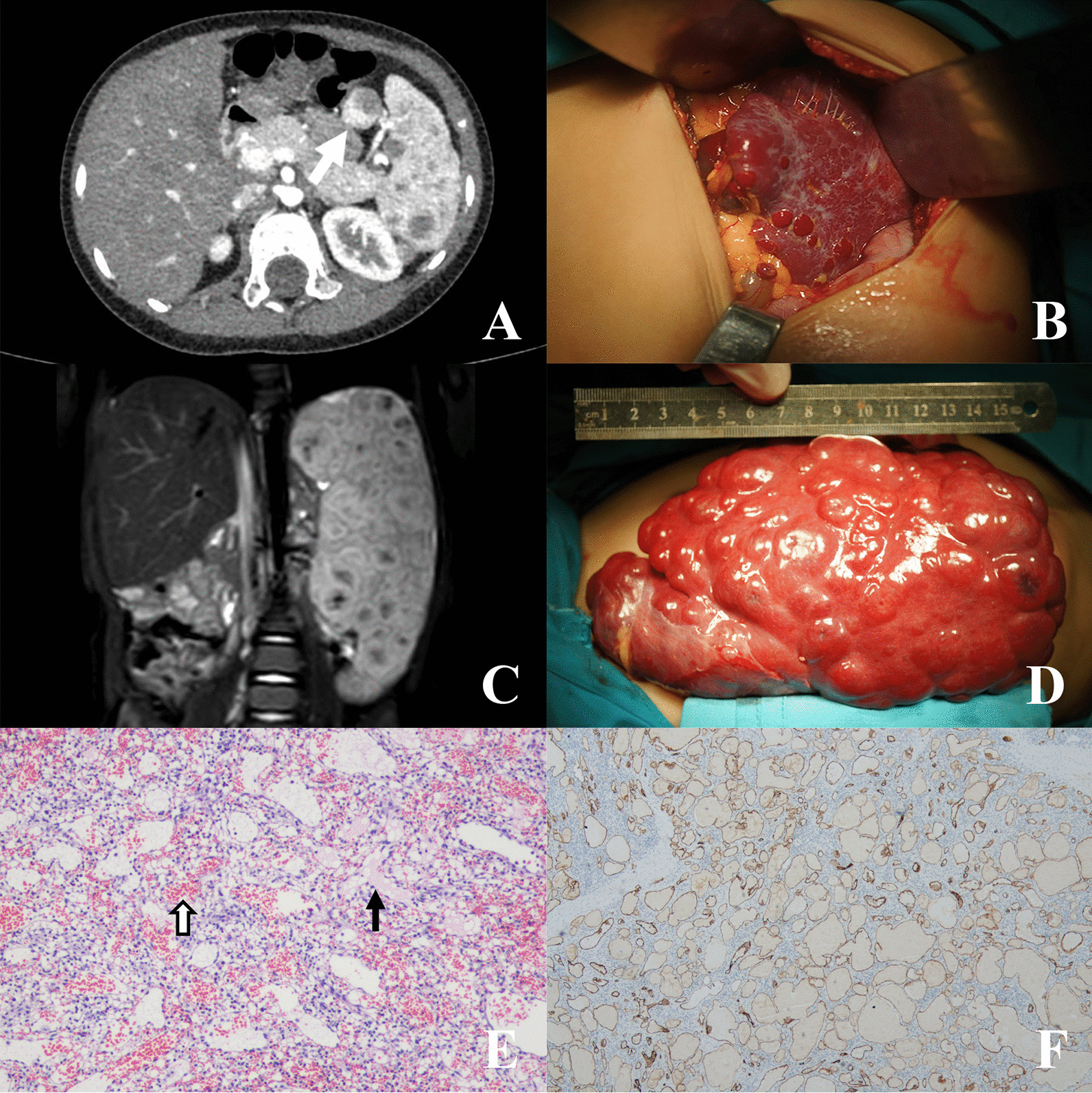
Splenic vascular malformations. **A** CT of splenic diffused lymphangiohemangiomatosis (white arrow shows the accessory spleen involved, Case 25). **B** Intraoperative picture of Case 25. **C** MRI of splenic diffused lymphangiomatosis (Case 20). **D** Intraoperative picture of Case 20. **E** HE staining of Case 25 shows lymphatic malformation space filled with eosinophilic amorphous proteinaceous fluid (black arrow) and capillary malformation space filled with blood (hollow arrow). **F** D2-40 immunohistochemical staining of Case 20 labels lymphatic endothelium

**Fig. 3:**
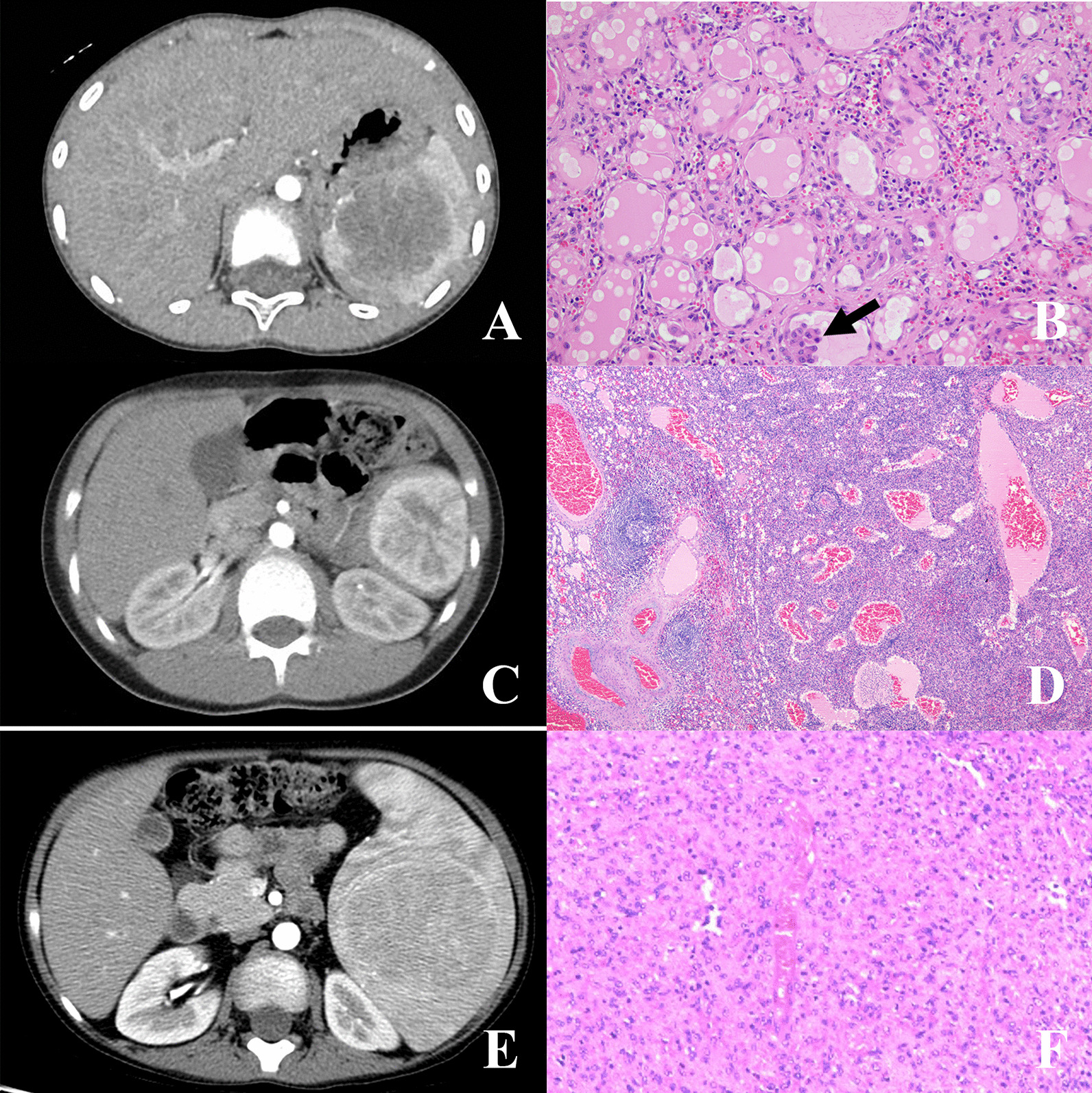
Imaging pictures (contrast CT) with corresponding pathology pictures (HE staining) of splenic focal lymphatic malformation, SANT, and splenic hamartoma. **A**, **B** Splenic focal lymphatic malformation (Case 30, black arrow shows focal endothelial papillary projections). **C**, **D**: SANT (Case 3, CT shows a typical “spoke-wheel” sign). **E**, **F**: Splenic hamartoma (Case 2, CT shows a heterogeneously enhanced hypodense mass)

Eight patients fell into this category, including 2 cases of isolated splenic diffused lymphangiohemangiomatosis (Fig. 2A, B), 1 isolated splenic diffused lymphangiomatosis (Fig. 2C, D), and 5 cases of splenic focal lymphatic malformation. In Case 25 the diffused lymphangiohemangiomatosis involved both the spleen and the accessory spleen (Fig. 2A). Microscopically lymphangiohemangiomatosis had both lymphatic and capillary malformation components (Fig. 2E, F). In case 30 the focal lymphatic malformation showed microscopically focal endothelial papillary projections without atypia (Fig. 3A, B). The patient (Case 19) with simultaneous separate CEC and hemangioma was not counted in, because his surgical indication was a CEC over 5 cm, not the hemangioma.

### SANT

Three patients fell into this category. They had typical histological features of SANT, including macroscopically red-brown nodules embedded in a dense fibrous stroma (typically a white stellate scar projecting towards the periphery from the center), microscopically individual nodules with angiomatoid appearance, and internodular stroma consisted of dense fibrous tissue (Fig. 3D). On imaging 2 patients (Case 3 and 8) showed typical spoke-wheel sign (Fig. 3C), and 1 (Case 6) did not.

### Hamartoma and leiomyoma

They are extremely rare in children, and 1 patient fell into each category. Microscopically the hamartoma (Case 2) showed disorganized vascular channels lined by slightly plump endothelial cells without white pulp (Fig. 3F), and Gamna-Gandy bodies could be seen occasionally. CT imaging showed a heterogeneously enhanced hypodense mass (Fig. 3E).

Microscopically the leiomyoma (Case 7) showed oval or spindle cells with focal vascular slit, and immunohistochemical staining showed: SMA + , Vimentin + , Ki-67 10% + , CD31 + (vascular slit), CD34 + (vascular slit). Unfortunately, the pathological slice and radiological image were unavailable for the leiomyoma patient.

### Lesion features and platelet counts changes

According the radiological images, surgical findings and pathological results, the location of the lesion in the spleen involved upper pole (7/30, 23.3%) hilum (6/30, 20.0%), lower pole (4/30, 13.3%), upper pole + hilum (7/30, 23.3%), lower pole + hilum (1/30, 3.3%), upper pole + hilum + lower pole (5/30, 16.7%).

All patients underwent open or laparoscopic surgery. Patients were divided into total splenectomy group and partial splenectomy group, and there were 15 patients in each group. Patients in the total splenectomy group are more likely to have a lesion involving the splenic hilum than those in the partial splenectomy group (68.4% vs 31.6%, P = 0.021, Fisher’s exact test). The 7-day postoperative platelet level was higher than the preoperative level in both total splenectomy group (mean difference 517.8 × 109/L, 95% CI 350.9 × 109/L-684.7 × 109/L, t = 6.655, P < 0.001) and partial splenectomy group (mean difference 180.6 × 109/L, 95% CI 101.2 × 109/L-260.0 × 109/L, t = 4.879, P < 0.001). In the analysis of covariance using preoperative platelet level as a covariate, the 7-day postoperative platelet level was higher in total splenectomy group than in partial splenectomy group (F = 11.97, P = 0.002, Table 2).

**Table 2:** Adjusted and unadjusted means and variability of 7-day postoperative platelet level in total and partial splenectomy patients with preoperative platelet level as a covariate

Group	N	Unadjusted (× 10^9^/L)		Adjusted* (× 10^9^/L)	
		Mean	SD	Mean	SE
Total splenectomy	15	694.0	266.7	694.4	58.7
Partial splenectomy	15	402.8	158.3	402.4	58.7

*N* number of patients, *SD* standard deviation, *SE* standard error

*F = 11.97, P = 0.002

## Discussion

Benign splenic lesions are rare with no accurate incidence reported. The incidence of focal splenic lesions on ultrasonography is about 0.1–0.2% [7]. A review of 42,327 autopsies found 32 cases of splenic cysts (0.075%) [8]. The most common lesions are cysts, followed by vascular malformations [3, 9]. As in our case series 56.7% (17/30) are CECs, and 26.7% (8/30) are vascular malformations. Some patients with benign splenic lesions are asymptomatic [10], however in surgical cases the most common clinical symptoms are abdominal pain in the left hypochondrium area, and abdominal mass (splenomegaly) [10, 11], which is in accordance with our surgical series. Our indications for surgical intervention include clinical symptoms, large lesions (> 5 cm), or diagnostic purposes, which were consistent with reported indications [12].

### Different epithelial linings of CECs support a metaplasia theory.

In children the most common type of nonparasitic splenic cysts is CEC [11], and we didn’t encounter any parasitic cysts, which might be geographically specific. The epithelial lining of CECs includes three basic types: mesothelium, transitional epithelium, and stratified squamous epithelium [13, 14]. These types can present in a single cyst or patient, and different epithelial linings can continue with each other [13], which are also true in our series. These phenomena support the metaplasia theory, that transitional and squamous epithelium originate from mesothelium metaplasia, perhaps because of the force and stretching from cyst expansion [13, 14]. CEC can partly or totally lose its epithelial lining under pressure degeneration [14, 15], and this was also found in 4 of our cases with squamous epithelium. In this situation, a single site pathological section finding no epithelial lining may lead to a misdiagnosis of pseudocyst [13].

### Diffused splenic vascular malformation can cause consumptive coagulopathy

According to the International Society for the Study of Vascular Anomalies (ISSVA), splenic lymphangioma and hemangioma should be considered as lymphatic and capillary malformations rather than tumors, and both belong to vascular malformations [11, 16]. A lesion with mixed form of lymphatic and capillary malformations is also proposed as “combined vascular malformation” by ISSVA classification [16, 17]. And this combined form was sometimes reported as lymphangiohemangioma [18]. When the splenic parenchyma is almost totally replaced by lymphatic or capillary malformations, it is called splenic lymphangiomatosis or hemangiomatosis [19, 20], or in a combined malformation it may be called “lymphangiohemangiomatosis”. Splenic large lymphangioma, especially lymphangiomatosis, can cause consumptive coagulopathy, usually chronic intravascular coagulopathy with elevated d-dimer ± mild to moderate thrombocytopenia [16, 17]. Both our cases of lymphangiohemagiomatosis (case 10 and 25) showed thrombocytopenia, with hypofibrinogenemia ± elevated d-dimer, suggesting sequestration of platelet in the lesion and consumptive coagulopathy[21]. The thrombocytopenia could intermittently improve spontaneously, which is distinct from Kasabach-Merritt Phenomenon (exclusively associated with the vascular tumors kaposiform hemangioendothelioma and tufted angioma, progressive and not expected to resolve spontaneously) [22].

### Extremely rare conditions do exist in pediatric benign splenic lesions

SANT is a non-neoplastic benign vascular lesion of the spleen first reported in 2004 by Martel [23]. Most reported cases are adults, but SANT can occur in children although very rare [24]. The macroscopic view of SANT shows multiple red-brown nodules scattered in a dense white fibrocalcific stroma, and the fibrous stroma often appears in a stellate form projecting from the center to the periphery [24]. On imaging (enhanced CT and MRI) some SANT can present a characteristic “spoke wheel” sign, as in two of our cases (Fig. 3C), which corresponds with the macroscopic central white stellate dense scar projecting to periphery [24, 25]. There are 3 distinct types of vessels in the angiomatoid nodules: CD34 + /CD8 − /CD31 + capillaries, CD34 − /CD8 + /CD31 + sinusoids, and CD34 − /CD8 − /CD31 + small veins, resembling the composition of normal splenic red pulp [23, 24]. These features are distinct from vascular malformation and endothelial neoplasm. Martel interpreted SANT as altered red pulp tissue that had been entrapped by a nonneoplastic stromal proliferative process, and they speculated that SANT might be a de novo lesion or the final common pathway of a variety of benign splenic conditions, including inflammatory pseudotumor, hamartoma, and hematoma [23]. SANT is totally benign and surgical removal (total or partial splenectomy) resulted in excellent prognosis with no recurrence in all reported cases [23, 24, 26–28].

Splenic hamartoma is a malformation composed of an anomalous mixture of normal splenic red pulp elements [29, 30]. It is very rare with a reported incidence of 0.024–0.13%, and even rarer in children [30, 31]. In our patient an obvious hemolytic process was observed with anemia and non-obstructive jaundice. The cordal meshwork of splenic red pulp can sensitively detect and phagocytose degenerating blood cells and foreign materials, which is referred to as the “culling function” [32]. In our patient the red blood cells might have been sequestrated and destructed in the hamartoma (abnormal red pulp) excessively (enhanced culling function of the hamartoma abnormal vascular channels), which is supported by the pathological presence of Gamna-Gandy body (small foci of hemosiderin deposits) [33, 34].

Splenic leiomyoma has been reported just in one case with ataxia-telangiectasia by Oguzkurt [35], and is extremely rare. Our case is the second reported splenic leiomyoma, and our patients did not have any other disease. We could only speculate that the leiomyoma might have originated from the smooth muscle cells of the tunica media of blood vessels. Ki-67 staining was 10% positive, and the lesion had an undetermined malignant potentiality. However, the patient remained uneventful after a follow up of 8 years.

## Surgical choices for pediatric benign splenic lesions

### Hold surgical indications strictly

Indications for surgical intervention should be considered carefully, which included clinical symptoms, large lesions (> 5 cm), or diagnostic purposes to exclude malignancy in our series. In our institution some patients were observed through outpatient without surgical indications, and some splenic hemangiomas were treated by interventional embolism. These patients were not discussed here but we stress to hold surgical indications strictly.

### Partial or total splenectomy may result in lower recurrence rate in CECs

Many surgical options for nonparasitic splenic cysts have been reported, including unroofing or fenestration of the cyst with or without inner surface coagulation, aspiration with or without sclerotherapy, and partial or total splenectomy [5]. Unroofing and aspiration cannot eliminate the epithelium completely, and was reported carrying a high recurrence rate [12, 36]. As in our series there were 3 recurrent patients who underwent a previous unroofing at other centers. No CEC patients showed recurrence after partial or total splenectomy in our series, and we recommend partial or total splenectomy as the first surgical option.

### Partial splenectomy is preferred over total splenectomy to preserve the splenic function

The spleen is the largest lymphatic organ of the human body, playing a crucial role in the immune and reticuloendothelial systems, modulating the inflammatory and coagulation cascades, and resisting encapsuled bacteria [32, 37]. Asplenic patients have increased risks of infections (including overwhelming post-splenectomy infection), arterial and venous thrombotic disease, pulmonary hypertension, and cancer [38]. Considering these important functions of the spleen and complications of asplenia, a spleen-preserving surgical strategy is highly recommended [39, 40].

Partial splenectomy is preferred to balance original disease recurrence and spleen preservation [41]. However, both the location of the lesion inside the spleen [42] and the splenic hilar vasculature type [40] may finally affect the spleen-preserving decision. A large, multifocal, hilar, or intra-parenchymal lesion, or a magistral supply hilar vasculature, may lead to a total splenectomy [40, 42]. In our series patients undergone total splenectomy are more likely to have a lesion involving the splenic hilum (68.4%) than those undergone partial splenectomy (31.6%).

Platelet number increases shortly after total or partial splenectomy [43], which can indirectly represent the degree of splenic function loss shortly after surgery. The mean 7-day postoperative platelet level is higher than the preoperative level in both partial and total splenectomy group, indicating a consequent splenic function decrease no matter which surgery performed. The adjusted mean platelet number (7-day after surgery) of partial splenectomy group (402.4 × 109/L) is lower than total splenectomy group (694.4 × 109/L), indicating an effective splenic function preservation in partial splenectomy group.

### Laparoscopic surgery is becoming first choice over open surgery

Recently minimally invasive surgery gained popularity, and laparoscopic/robotic partial or total splenectomy are becoming preferred options [5, 6, 39, 44]. In our institution laparoscopic surgery is becoming first choice in recent years, which can be seen in Table 1, that from case 1 to case 30 (ranging in chronological order) more laparoscopic surgeries were carried out. However, surgeons performing minimally invasive splenic surgery should not underestimate its technical difficulty and complexity [39]. Conversion to open procedure is necessary in case of massive bleeding or inconvenient manipulation, to avoid large inner trauma under smaller incisions [39, 44].

## Limitations

Due to the retrospective nature of this study and the long span of time, we were unable to get some of the radiological images and pathological slices, but information was still partly available on electronic scanned paper reports. We didn’t perform any other treatments on CECs, such as unroofing or sclerotherapy, as a result we could provide any related data and experience. Minimally invasive surgeries were more commonly performed in recent years than in early years, yet we cannot get any conclusions about its advantages or disadvantages, considering many confounding factors, such as different surgeons, learning curve, various disease spectrum, and limited patient number. At last, the long-term residual spleen filtration function was not measured, which can be measured by the detection of red cells with Howell-Jolly bodies and liver-spleen scintigraphy scanning.

## Conclusions

In conclusion, pediatric benign splenic lesions are rare, and in this study, we summarized various clinical characteristics of this rare entity. The most common pathological diagnoses are congenital epithelial cyst and vascular malformation. Partial and total splenectomy result in good prognosis with a low recurrence rate, and the former is preferred to preserve splenic function if possible.

## Data Availability

The data and materials used and/or analyzed during the current study are available from the corresponding author on reasonable request.

## Abbreviations

CEC: Congenital epithelial cyst
SANT: Sclerosing angiomatoid nodular transformation
ISSVA: International Society for the Study of Vascular Anomalies
